# Comparative Performance Evaluation of the Public Health Surveillance Systems in 6 Gulf Cooperation Countries: Cross-sectional Study

**DOI:** 10.2196/41269

**Published:** 2023-04-05

**Authors:** Nawaf Albali, Sami Almudarra, Yahya Al-Farsi, Abdullah Alarifi, Adil Al Wahaibi, Pasi Penttinen

**Affiliations:** 1 Health & Public Sector Accenture Saudi Arabia Riyadh Saudi Arabia; 2 Gulf Center of Disease Prevention and Control Gulf Health Council Riyadh Saudi Arabia; 3 Department of Family Medicine and Public Health College of Medicine and Health Sciences Sultan Qaboos University Muscat Oman; 4 Health Research Unit Gulf Health Council Riyadh Saudi Arabia; 5 Department of Public Health College of Health Sciences Saudi Electronic University Riyadh Saudi Arabia; 6 Department of Surveillance Ministry of Health Muscat Oman

**Keywords:** Gulf Cooperation Council, surveillance systems, program evaluation, performance evaluation, disease monitoring, emerging technologies

## Abstract

**Background:**

Evaluating public health surveillance systems is important to ensure that events of public health importance are appropriately monitored. Evaluation studies based on the Centers for Disease Control and Prevention (CDC) guidelines have been used to appraise surveillance systems globally. Previous evaluation studies undertaken in member countries of the Gulf Cooperation Council (GCC) were limited to specific illnesses within a single nation.

**Objective:**

We aimed to evaluate public health surveillance systems in GCC countries using CDC guidelines and recommend necessary improvements to enhance these systems.

**Methods:**

The CDC guidelines were used for evaluating the surveillance systems in GCC countries. A total of 6 representatives from GCC countries were asked to rate 43 indicators across the systems’ level of usefulness, simplicity, flexibility, acceptability, sensitivity, predictive value positive, representativeness, data quality, stability, and timeliness. Descriptive data analysis and univariate linear regression analysis were performed.

**Results:**

All surveillance systems in the GCC covered communicable diseases, and approximately two-thirds (4/6, 67%, 95% CI 29.9%-90.3%) of them covered health care–associated infections. The mean global score was 147 (SD 13.27). The United Arab Emirates scored the highest in the global score with a rating of 167 (83.5%, 95% CI 77.7%-88.0%), and Oman obtained the highest scores for usefulness, simplicity, and flexibility. Strong correlations were observed between the global score and the level of usefulness, flexibility, acceptability, representativeness, and timeliness, and a negative correlation was observed between stability and timeliness score. Disease coverage was the most substantial predictor of the GCC surveillance global score.

**Conclusions:**

GCC surveillance systems are performing optimally and have shown beneficial outcomes. GCC countries must use the lessons learned from the success of the systems of the United Arab Emirates and Oman. To maintain GCC surveillance systems so that they are viable and adaptable to future potential health risks, measures including centralized information exchange, deployment of emerging technologies, and system architecture reform are necessary.

## Introduction

### Background

Evaluating public health surveillance systems is critical to ensure that events of public health importance are appropriately monitored. The US Centers for Disease Control and Prevention’s (CDC) original Guidelines for Evaluating Surveillance Systems, published in 1988 and updated in 2001, emphasize the need for prioritizing the use of available resources to maximize efficiency and promote best practices in surveillance monitoring [1].

Increased concern regarding bioterrorism and the embryonic development of early outbreak detection technologies prompted the issuance of the Framework for Evaluating Public Health Surveillance Systems for Early Detection of Outbreaks in 2004 [2]. The framework leverages CDC’s current evaluation guidelines by emphasizing the need to evaluate all system attributes, including the level of usefulness, simplicity, flexibility, acceptability, sensitivity, predictive value positive (PVP), representativeness, data quality, stability, and timeliness, including syndromic surveillance systems.

The Gulf Cooperation Council (GCC) is a coalition of 6 Arab countries that have similar socioeconomic characteristics and political outlooks: the United Arab Emirates (UAE), Bahrain, Saudi Arabia, Oman, Qatar, and Kuwait. All 6 nations are members of the Gulf Health Council, the executive arm of the Council of Ministers of Health in the GCC region.

Surveillance systems evaluation attained paramount importance following the establishment of the Gulf Center of Disease Prevention and Control (Gulf CDC) in 2021, with the approval of Their Majesties and Their Highnesses, Leaders of the 6 Gulf Cooperation States [3]. A core mandate for this entity was the fostering of harmonization in public health data and surveillance efforts through systematic and ongoing surveillance evaluation, as well as the proper use of public health data exchange practices. The Gulf CDC is currently housed within the Gulf Health Council.

Public health surveillance systems in the GCC region have witnessed considerable growth and maturity over the last decade, and the GCC has been widely lauded for ensuring adequate health surveillance systems [4]. However, this growth has been accompanied by an increase in the communicable disease (CD) and noncommunicable disease (NCD) burden, and there is a scarcity of evidence to evaluate the systems’ effectiveness in responding to new disease patterns and shifting population characteristics [5]. Therefore, it is crucial to generate and disseminate new knowledge with regard to surveillance systems in GCC countries to assess the systems’ utility, effectiveness, and health outcomes.

Evaluation studies using CDC guidelines have been used to assess surveillance systems in different regions of the world and for several health-related events [6,7]. Despite the known maturity of health surveillance system performance worldwide, there is a lack of empirical research evaluating public health surveillance systems in the GCC region. The few studies undertaken in the GCC have focused on specific diseases within a single country; moreover, limited studies have focused on system attributes according to the CDC’s Public Health Surveillance Evaluation Guidelines [8,9].

### Objectives

We aimed to evaluate public health surveillance systems in the GCC regarding their level of usefulness, simplicity, flexibility, acceptability, sensitivity, PVP, representativeness, data quality, stability, and timeliness. The specific objectives were as follows: (1) compare the characteristics of selected public health surveillance systems across GCC countries, (2) estimate the performance scores of GCC public health surveillance systems based on CDC surveillance evaluation attributes, and (3) explore the association between performance scores and GCC surveillance systems characteristics.

## Methods

### Study Design and Population Characteristics

A cross-sectional evaluation study was conducted to assess the GCC surveillance systems and their system attributes by using the CDC guidelines for evaluating disease surveillance systems. This study was conducted in Riyadh City from March to June 2022. The evaluation included an assessment of the usefulness of the system and other system attributes including simplicity, flexibility, acceptability, sensitivity, PVP, representativeness, data quality, stability, and timeliness. These characteristics were of importance for the hardware and software, the standard user interface, standard data format and coding, proper quality checks, and adherence to confidentiality and security requirements.

Participation was voluntary, and the inclusion criteria were senior surveillance administrators with considerable experience and knowledge in implementing nationwide surveillance programs across GCC countries.

### Selection of Country Representatives

The authors coordinated with the Gulf CDC to identify 6 country representatives who could respond on behalf of their countries using the purposive sampling technique. Within the Gulf CDC’s governance structure, the Gulf CDC Supervisory Committee acts as the Center’s Board of Directors. Inclusion criteria mandated that the country representative must be an epidemiologist or a public health professional with >5 years of experience in managing national-level surveillance systems. According to the inclusion criteria, the questionnaire was submitted to Supervisory Committee members, who purposively identified the appropriate representative to respond to each country. This technique enabled the accurate selection of representatives for each country and aided in preventing selection bias [10].

### Study Tools

A standardized self-reported questionnaire with defined indicators to assess system attributes was developed. The indicators were created based on the CDC guidelines for evaluating disease surveillance systems and by using a review of prior public health surveillance assessments. The questions were constructed by 3 experts with experience in the establishment and assessment of surveillance systems, and content validity was determined by a panel of 5 epidemiologists, public health professionals, and preventive medicine specialists. The questionnaire was validated by 2 representatives from GCC member countries, and these responses were not included in the main research. Only minor adjustments were made to the original questions to improve clarity. The number of indicators used varied depending on the attributes assessed. Participants were asked to assess their agreement with the attributes’ exact indicators on a 5-point Likert scale (1=strongly disagree, 2=disagree, 3=neutral, 4=agree, and 5=strongly agree), with a higher score representing better performance on the assessed attribute.

### Operational Definitions of System Attributes

#### Level of Usefulness

The country representatives were prompted to score 10 indicators of usefulness on a 5-point Likert scale. The system was deemed useful if it could produce estimates of morbidity and mortality rates, identify high-risk populations and the social determinants of fatalities, generate actionable reports and insights instantly, permit the evaluation of the impact of preventive and control programs, anticipate trends and outcomes using artificial intelligence (AI) and machine learning models, and stimulate research.

#### Simplicity

The system’s simplicity was assessed in terms of both its structure and ease of use. A total of 6 indicators of simplicity were evaluated by the representatives. The indicators evaluated the clarity of the case definitions, the requirement for regular training, the convenience of acquiring data, and the country’s integration level with the health information system.

#### Flexibility

Flexibility alludes to the system’s ability to handle new health-related events and case definition revisions and its ease of integration with other systems. The representatives evaluated 3 indicators including the ability to respond quickly to a new demand, such as diseases and risk factors; the ability to respond quickly to emerging and re-emerging pandemics; and the ability to detect events other than notifiable diseases, such as food poisoning, chemical poisoning, and environmental events.

#### Acceptability

Representatives from each country evaluated 3 indicators of acceptability including the completion rates of entered forms, the associated laboratory completion rates, and the completion rates of hospitals and primary health centers.

#### Sensitivity and PVP

Neither the authors nor the representatives from the countries statistically evaluated the positive attributes of sensitivity and predictive value because of the heterogeneity of diseases covered in each country’s surveillance system. Therefore, representatives were required to rate 4 indicators of sensitivity and predictive values as proxies for the actual calculation. Indicators included the system’s ability to correctly detect a high proportion of disease cases and new epidemics or outbreaks, the proportion of reported cases that are true cases, and the proportion of reported epidemics that are true epidemics.

#### Representativeness

Four indicators were used to assess the accuracy of the surveillance systems in describing the occurrence of a health-related event over time and their distribution in the population by place and person. In addition, the system’s ability to measure the natural history of the disease, store clinical practice data (sites performing diagnostic tests and physician-referral patterns), and store clinical outcomes of interest (mortality data, hospitalizations, and laboratory data) was assessed.

#### Data Quality

The data quality reflects the completeness and validity of the data recorded in each country’s public health surveillance system. The representatives rated 3 indicators of data quality in their respective country including the accuracy of the data entered, presence of standardized methods for data entry, and internal validity of the surveillance system’s data.

#### Stability

Stability refers to the reliability (ie, the ability to collect, manage, and provide data without failure) and availability (the ability to operate when needed) of the public health surveillance system. Four indicators were used to evaluate the stability. The indicators assessed spontaneous outages and downtimes, resources dedicated to technical repairs to maintain system functionality, and the time required for gathering, receiving, and managing data.

#### Timeliness

Timeliness refers to the rapid availability of sufficient data to enable public health authorities to take appropriate actions. Two indicators were developed and assessed for timeliness, including the system’s ability to rapidly generate data for immediate disease control and long-term program planning.

### Data Analysis

The indicators assessing system attributes were scored on a Likert scale from 1 (“strongly disagree”) to 5 (“strongly agree”). Independent variables, including attribute scores for each country, were calculated as the sum of each indicator’s scores. The global scores for each country were then calculated as the sum of the attribute scores for that specific country. The dependent variables were the types of diseases covered by the surveillance systems, their data storage type, the presence of syndromic surveillance, and their placement within the original system.

Differences among the various proportions of categorical data were evaluated for statistical significance using the chi-square test. Fisher exact test (2-tailed) was used to counteract the small sample size, where the expected frequency was <5 in any of the cells in the 2 × 2 tables. A nonparametric test (Mann-Whitney) was used to ascertain substantial differences when the assumption of normality was violated. Continuous variables were represented as mean and SD. Episheets software was used to calculate the CIs [11]. Descriptive data analysis and depiction of graphs were performed using Microsoft Excel (Microsoft Office 365).

For all statistical tests, a *P* value ≤.05 was considered statistically significant. All statistical analyses were performed using SPSS software (version 24.0; IBM Corp). A univariate linear regression analysis was conducted to confirm the effects of various systems characteristics on the global score.

### Ethics Approval

Ethics approval for this study was obtained from the Institutional Review Committee in King Fahad Medical City, Ministry of Health, Saudi Arabia (Institutional Review Board Log Number: 21-511E).

### Informed Consent and Data Integrity

A written informed consent was obtained from the representatives before their participation. Representatives were informed that they were free to refuse to participate and withdraw from the study at any time without any disadvantage or prejudice. Confidentiality and privacy were ensured by assigning a code for each country. They were notified about their rights regarding being informed about the way their data would be used and that it would be used only after ensuring that it would not cause harm. No compensation was provided to the participants for their involvement in this research; participants voluntarily agreed to engage in the study to contribute to the research and advance their knowledge in the field.

## Results

### Surveillance Systems Characteristics

Table 1 shows the selected characteristics of public health surveillance systems across GCC countries. All surveillance systems in the GCC covered CDs, and approximately two-thirds of them covered health care–associated infections (HAIs; 4/6, 67%, 95% CI 29.9%-90.3%). Three countries reported having full electronic surveillance systems, whereas the others reported that their systems were a hybrid between electronic and paper-based operations (3/6, 50%, 95% CI 18.7%-81.2%). The use of on-premises data storage was reported in 4 countries, compared with 2 countries that stored the surveillance data on-cloud storage (4/6, 67%, 95% CI 29.9%-90.3%). While all GCC countries reported having syndromic surveillance, 1 country reported that its syndromic surveillance system was separate from the original system (1/6, 17%, 95% CI 0.80%-59.0%).

Table 2 details select characteristics of the public health surveillance for each country. The UAE reported covering all diseases of interest including CDs, NCDs, injuries and falls, HAIs, and environmental and occupational diseases. The Sultanate of Oman’s surveillance system covered CDs, HAIs, and environmental and occupational diseases. CDs and environmental and occupational diseases were covered in the State of Qatar. The surveillance systems of both Bahrain and Saudi Arabia covered CDs and HAIs. However, the surveillance system in Kuwait only covered CDs.

**Table 1:** Selected public health surveillance characteristics among Gulf Cooperation Council member states (N=6).

System characteristics		Total, n (%, 95% CI)
**Disease coverage**		
	CDs^a^	6 (100, 60.9-100)
	NCDs^b^	1 (17, 0.80-59)
	Injuries and falls	1 (17, 0.80-59)
	HAIs^c^	4 (67, 29.9-90.3)
	EODs^d^	3 (50, 18.7-81.2)
**System type**		
	Electronic	3 (50, 18.7-81.2)
	Hybrid	3 (50, 18.7-81.2)
**Storage type**		
	On premises	4 (67, 29.9-90.3)
	On cloud	2 (33, 9.6-70)
**Syndromic surveillance**		
	Yes	6 (100, 60.9-100)
**Place of SS^e^**		
	Within the original system	5 (83, 43.6-96.9)
	Separate from the original system	1 (17, 0.80-59)

^a^CD: communicable disease.

^b^NCD: noncommunicable disease.

^c^HAI: health care–associated infection.

^d^EOD: environmental and occupational disease.

^e^SS: syndromic surveillance.

**Table 2:** Selected public health surveillance characteristics among Gulf Cooperation Council member states, by country.

System characteristics			Total across all countries (N=6), n (%)		Saudi Arabia		Kuwait		United Arab Emirates		Oman		Qatar		Bahrain
**Disease coverage**															
	CDs^a^	6 (100)		Present		Present		Present		Present		Present		Present	
	NCDs^b^	1 (16)		Absent		Absent		Present		Absent		Absent		Present	
	Injuries and falls	1 (16)		Absent		Absent		Present		Absent		Absent		Absent	
	HAIs^c^	4 (66)		Present		Absent		Present		Present		Absent		Present	
	EODs^d^	3 (50)		Absent		Absent		Present		Present		Present		Absent	
	Disease coverage within each country (n=5), n (%)	—^e^		2 (40)		1 (20)		5 (100)		3 (60)		2 (40)		2 (40)	
**System type**															
	Electronic	3 (50)		Present		Absent		Absent		Present		Present		Absent	
	Paper-based	0 (0)		Absent		Absent		Absent		Absent		Absent		Absent	
	Hybrid	3 (50)		Absent		Present		Present		Absent		Absent		Present	
**Storage type**															
	On premises	4 (66)		Absent		Present		Present		Absent		Present		Present	
	On cloud	2 (33)		Present		Absent		Absent		Present		Absent		Absent	
**Syndromic surveillance**															
	Yes	6 (100)		Present		Present		Present		Present		Present		Present	
	No	0 (0)		Absent		Absent		Absent		Absent		Absent		Absent	
**Place of SS^f^**															
	Within the original system	5 (83)		Present		Present		Present		Present		Absent		Present	
	Separate from the original system	1 (16)		Absent		Absent		Absent		Absent		Present		Absent	

^a^CD: communicable disease.

^b^NCD: noncommunicable disease.

^c^HAI: health care–associated infection.

^d^EOD: environmental and occupational disease.

^e^Not applicable.

^f^SS: syndromic surveillance.

From a system-type perspective, Saudi Arabia, Qatar, and Oman reported having fully electronic surveillance systems, whereas Bahrain, Kuwait, and the UAE reported having hybrid surveillance systems. Moreover, Bahrain, Kuwait, the UAE, Oman, and Qatar stored their surveillance data using on-premises servers, whereas Oman and Saudi Arabia used on-cloud data storage. Generally, surveillance systems in the GCC regions had syndromic surveillance embedded with the original systems; however, the State of Qatar was an exception, as their syndromic surveillance was separate from the original system.

### Surveillance Systems Evaluation

**Figure 1:**
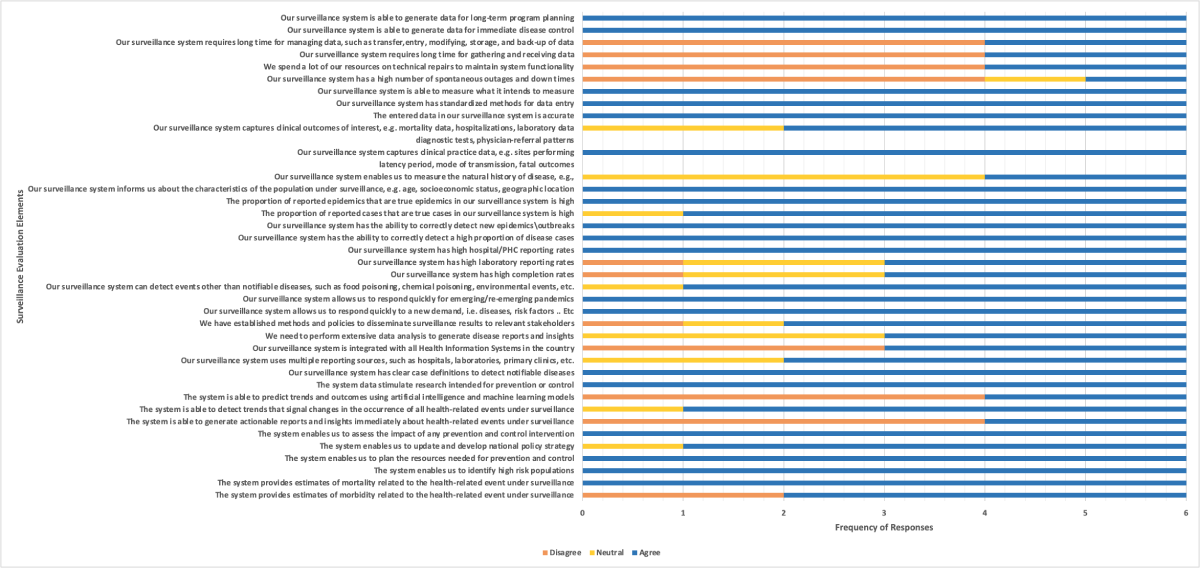
Responses to individual questions in the Centers for Disease Control and Prevention (CDC) surveillance evaluation framework over selected Likert scale categories (agree, neutral, and disagree). PHC: primary health care center.

Table 3 shows the frequency distribution of participants’ responses to each question in the questionnaire for all 5 Likert scale categories. Figure 1 shows the frequency of the participants' responses to each question in the questionnaire over the 3 collapsed categories: agree, neutral, and disagree. Only 2 countries used new cutting-edge technologies, resulting in a glaring mismatch among the countries in the use of machine learning, AI, and real-time analytics.

**Table 3:** Responses to individual questions in the Centers for Disease Control and Prevention surveillance evaluation framework over all Likert scale categories (N=6).

Question	Strongly disagree, n (%)	Disagree, n (%)	Neutral, n (%)	Agree, n (%)	Strongly agree, n (%)
The system provides estimates of morbidity related to the health-related event under surveillance	1 (17)	1 (17)	0 (0)	4 (67)	0 (0)
The system provides estimates of mortality related to the health-related event under surveillance	0 (0)	0 (0)	0 (0)	5 (83)	1 (17)
The system enables us to identify high-risk populations	0 (0)	0 (0)	0 (0)	4 (67)	2 (33)
The system enables us to plan the resources needed for prevention and control	0 (0)	0 (0)	0 (0)	3 (50)	3 (50)
The system enables us to update and develop national policy strategy	0 (0)	0 (0)	1 (17)	2 (33)	3 (50)
The system enables us to assess the impact of any prevention and control intervention	0 (0)	0 (0)	0 (0)	5 (83)	1 (17)
The system can generate actionable reports and insights immediately about health-related events under surveillance	0 (0)	4 (67)	0 (0)	0 (0)	2 (33)
The system can detect trends that signal changes in the occurrence of all health-related events under surveillance	0 (0)	0 (0)	1 (17)	3 (50)	2 (33)
The system can predict trends and outcomes using artificial intelligence and machine learning models	1 (17)	3 (50)	0 (0)	1 (17)	1 (17)
The system data stimulate research intended for prevention or control	0 (0)	0 (0)	0 (0)	3 (50)	3 (50)
Our surveillance system has clear case definitions to detect notifiable diseases	0 (0)	0 (0)	0 (0)	3 (50)	3 (50)
Our surveillance system uses multiple reporting sources, such as hospitals, laboratories, primary clinics, etc.	0 (0)	0 (0)	2 (33)	1 (17)	3 (50)
Our surveillance system is integrated with all health information systems in the country	1 (17)	2 (33)	0 (0)	1 (17)	2 (33)
We need to perform extensive data analysis to generate disease reports and insights	0 (0)	0 (0)	3 (50)	2 (33)	1 (17)
We have established methods and policies to disseminate surveillance results to relevant stakeholders	0 (0)	1 (17)	1 (17)	2 (33)	2 (33)
Our surveillance system allows us to respond quickly to a new demand, that is, diseases, risk factors etc.	0 (0)	0 (0)	0 (0)	3 (50)	3 (50)
Our surveillance system allows us to respond quickly for emerging or re-emerging pandemics	0 (0)	0 (0)	0 (0)	3 (50)	3 (50)
Our surveillance system can detect events other than notifiable diseases, such as food poisoning, chemical poisoning, environmental events, etc.	0 (0)	0 (0)	1 (17)	2 (33)	3 (50)
Our surveillance system has high completion rates	0 (0)	1 (17)	2 (33)	3 (50)	0 (0)
Our surveillance system has high laboratory reporting rates	0 (0)	1 (17)	2 (33)	3 (33)	0 (0)
Our surveillance system has high hospital or PHC^a^ reporting rates	0 (0)	0 (0)	0 (0)	5 (83)	1 (17)
Our surveillance system can correctly detect a high proportion of disease cases	0 (0)	0 (0)	0 (0)	5 (83)	1 (17)
Our surveillance system can correctly detect new epidemics or outbreaks	0 (0)	0 (0)	0 (0)	5 (83)	1 (17)
The proportion of reported cases that are true cases in our surveillance system is high	0 (0)	0 (0)	1 (17)	3 (50)	2 (33)
The proportion of reported epidemics that are true epidemics in our surveillance system is high	0 (0)	0 (0)	0 (0)	4 (67)	2 (33)
Our surveillance system informs us about the characteristics of the population under surveillance, for example, age, socioeconomic status, geographic location	0 (0)	0 (0)	0 (0)	4 (67)	2 (33)
Our surveillance system enables us to measure the natural history of disease, for example, latency period, mode of transmission, and fatal outcomes	0 (0)	0 (0)	4 (67)	1 (17)	1 (17)
Our surveillance system captures clinical practice data, for example, sites performing diagnostic tests, and physician-referral patterns	0 (0)	0 (0)	0 (0)	5 (83)	1 (17)
Our surveillance system captures clinical outcomes of interest, for example, mortality data, hospitalizations, and laboratory data	0 (0)	0 (0)	2 (33)	2 (33)	2 (33)
The entered data in our surveillance system is accurate	0 (0)	0 (0)	0 (0)	1 (17)	5 (83)
Our surveillance system has standardized methods for data entry	0 (0)	0 (0)	0 (0)	3 (50)	3 (50)
Our surveillance system can measure what it intends to measure	0 (0)	0 (0)	0 (0)	4 (67)	2 (33)
Our surveillance system has a high number of spontaneous outages and down times	1 (17)	3 (50)	1 (17)	1 (17)	0 (0)
We spend a lot of our resources on technical repairs to maintain system functionality	1 (17)	3 (50)	0 (0)	2 (33)	0 (0)
Our surveillance system requires a long time for gathering and receiving data	1 (17)	3 (50)	0 (0)	2 (33)	0 (0)
Our surveillance system requires a long time for managing data, such as transfer, entry, modifying, storage, and back-up of data	2 (33)	2 (33)	0 (0)	1 (17)	1 (17)
Our surveillance system is able to generate data for immediate disease control	0 (0)	0 (0)	0 (0)	4 (67)	2 (33)
Our surveillance system is able to generate data for long-term program planning	0 (0)	0 (0)	0 (0)	4 (67)	2 (33)

^a^PHC: primary health care center.

Table 4 illustrates the means of global and attribute scores of the GCC surveillance systems. Overall, the mean global score was 147 (SD 13.27). The means for usefulness, simplicity, flexibility, acceptability, sensitivity, PVP, representativeness, data quality, stability, and timeliness were 39, 19.3, 13.3, 10.8, 8.3, 8.5, 16, 13, 9.8, and 8.6, respectively.

**Table 4:** Descriptive statistics of the Gulf Cooperation Council surveillance systems for global and attribute scores (N=6).

System attributes	Values, mean (SD)	Values, median (IQR; range)
Global score	147 (13.2)	144 (25.75; 133-167)
Level of usefulness	39 (5.1)	36.5 (10; 34-46)
Simplicity	19.3 (4.3)	18 (9; 15-25)
Flexibility	13.3 (1.3)	13 (3; 12-15)
Acceptability	10.8 (1.7)	11 (3; 9-13)
Sensitivity	8.3 (0.81)	8 (1; 8-10)
Predictive value positive	8.5 (1.2)	8 (2; 7-10)
Representativeness	16 (2.4)	15 (5;14-20)
Data quality	13 (1.2)	12.5 (2; 12-15)
Stability	9.8 (3.1)	10 (5; 5-14)
Timeliness	8.6 (1)	8 (2; 8-10)

Table 5 depicts the scores of the GCC Surveillance Systems’ Global and Attribute Scores by country. The UAE scored the highest among the GCC countries in terms of the global score, with a score of 167 (83.5%, 95% CI 77.7%-88.0%). On the attribute level, Oman scored the highest on usefulness, simplicity, and flexibility, scoring 46 (92%, 95% CI 81.1%-96.8%), 25 (71.4%, 95% CI 54.9%-83.6%), and 15 (100%, 95% CI 79.6%-100%), respectively. However, the UAE scored the highest on acceptability, sensitivity, PVP, representativeness, data quality, and timeliness, whereas Qatar scored the highest on stability, obtaining a score of 10 (50%, 95% CI 28.8%-71.1%).

**Table 5:** Scores of Gulf Cooperation Council surveillance systems global and attribute scores by country (N=6).

Attributes	Saudi Arabia			Kuwait			United Arab Emirates			Oman			Qatar			Bahrain	
	Score	Proportion (95% CI)	Score		Proportion (95% CI)	Score		Proportion (95% CI)	Score		Proportion (95% CI)	Score		Proportion (95% CI)	Score		Proportion (95% CI)
Global score	145	72.5 (65.9-78.2)	133		66.5 (59.7-72.6)	167		83.5 (77.7-88.0)	158		79 (72.8-84.0)	143		71.5 (64.8-77.3)	135		67.5 (60.7-73.6)
Usefulness	37	74 (60.4-84.1)	36		72.0 (58.3-82.5)	45		90 (78.6-95.6)	46		92 (81.1-96.8)	36		72 (58.3-82.5)	34		68 (54.1-79.2)
Simplicity	15	42.8 (27.9-59.1)	16		45.7 (30.4-61.8)	24		68.5 (52.0-81.4)	25		71.4 (54.9-83.6)	20		57.1 (40.8-72)	16		45.7 (30.4-61.8)
Flexibility	13	86.6 (62.1-96.2)	12		80.0 (54.8-92.9)	15		100 (79.6-100)	15		100 (79.6-100)	13		86.6 (62.1-96.2)	12		80.0 (54.8-92.9)
Acceptability	12	80.0 (54.8-92.9)	9		60.0 (35.7-80.1)	13		86.6 (62.1-96.2)	12		80.0 (54.8-92.9)	10		66.7 (41.7-84.8)	9		60.0 (35.7-80.1)
Sensitivity	8	80.0 (49.0-94.3)	8		80.0 (49.0-94.3)	10		100 (72.26-100)	8		80.0 (49.0-94.3)	8		80.0 (49.0-94.3)	8		80.0 (49.0-94.3)
Predictive value positive	8	80.0 (49.0-94.3)	8		80.0 (49.0-94.3)	10		100 (72.26-100)	7		70.0 (39.6-89.2)	8		80.0 (49.0-94.3)	8		80.0 (49.0-94.3)
Representative	18	90.0 (69.9-97.2)	14		70.0 (48.1-85.4)	20		100 (83.8-100.0)	15		75.0 (52.9-90.2)	15		75.0 (52.9-90.2)	14		70.0 (48.1-86.8)
Data quality	14	93.3 (71.2-99.6)	12		80.0 (54.6-94.6)	15		100.0 (81.8-100.0)	12		80.0 (54.6-94.6)	13		86.6 (62.4-97.7)	12		80.0 (54.6-94.6)
Stability	12	60.0 (37.8-79.4)	10		50.0 (28.8-71.1)	5		25.0 (9.7-47.0)	8		40.0 (20.6-62.1)	10		50.0 (28.8-71.1)	14		70.0 (48.1-85.4)
Timeliness	8	80.0 (49.0-94.3)	8		80.0 (49.0-94.3)	10		100 (72.26-100)	10		100 (72.26-100)	8		80.0 (49.0-94.3)	8		80.0 (49.0-94.3)

Table 6 shows the correlation coefficients (*r*) between the global score and each of the attribute scores of the GCC surveillance systems. Generally, significant strong positive correlations were obtained between the global score and the following attributes: level of usefulness (*r*=0.841; *P*=.036), flexibility (*r*=0.956; *P*=.003), acceptability (*r*=0.971; *P*=.001), representativeness (*r*=0.883, *P*=.020), and timeliness (*r*=0.828; *P*=.042). Strong and positive correlations were obtained between the level of usefulness and flexibility (*r*=0.910; P=.012), acceptability (*r*=0.851; *P*=.032), and timeliness (*r*=0.840; *P*=.036), which were statistically significant. Moreover, strong correlations were found between flexibility and acceptability (r=0.923; *P*=.009) and between acceptability and representativeness (*r*=0.955; *P*=.003). The correlation between stability and timeliness scores was negative, strong, and statistically significant (*r*=−0.840; *P*=.036).

**Table 6:** Interitem correlation matrix between global and attribute scores of Gulf Cooperation Council surveillance systems.

Variable		Global	USE^a^	SIM^b^	FLE^c^	ACC^d^	SEN^e^	PVP^f^	REP^g^	DQ^h^	STB^i^	TIM^j^
**Global**												
	*r*	1.000	—^k^	—	—	—	—	—	—	—	—	—
	*P* value	—	—	—	—	—	—	—	—	—	—	—
**USE**												
	*r*	0.841^l^	1.000	—	—	—	—	—	—	—	—	—
	*P* value	.036	—	—	—	—	—	—	—	—	—	—
**SIM**												
	*r*	0.580	0.603	1.000	—	—	—	—	—	—	—	—
	*P* value	.228	.205	—	—	—	—	—	—	—	—	—
**FLE**												
	*r*	0.956^m^	0.910^l^	0.728	1.000	—	—	—	—	—	—	—
	*P* value	.003	.012	.101	—	—	—	—	—	—	—	—
**ACC**												
	*r*	0.971^m^	0.851^l^	0.448	0.923^m^	1.000	—	—	—	—	—	—
	*P* value	.001	.032	.009	.009	—	—	—	—	—	—	—
**SEN**												
	*r*	0.655	0.399	0.399	0.548	0.674	1.000	—	—	—	—	—
	*P* value	.158	.434	.434	.261	.142	—	—	—	—	—	—
**PVP**												
	*r*	0.123	−0.235	0.000	0.065	0.191	0.566	1.000	—	—	—	—
	*P* value	.816	.654	1.000	.903	.717	.242	—	—	—	—	—
**REP**												
	*r*	0.883^l^	0.687	0.239	0.800	0.955^m^	0.674	0.397	1.000	—	—	—
	*P* value	.020	.132	.649	.056	.003	.142	.435	—	—	—	—
**DQ**												
	*r*	0.638	0.339	−0.031	0.508	0.750	0.696	0.689	0.907^l^	1.000	—	—
	*P* value	.173	.511	.954	.304	.066	.125	.130	.013	—	—	—
**STB**												
	*r*	−0.667	−0.765	−0.809	−0.788	−0.672	−0.664	−0.204	−0.537	−0.339	1.000	—
	*P* value	.148	.077	.051	.063	.144	.150	.699	.272	.511	—	—
**TIM**												
	*r*	0.828^l^	0.840^l^	0.840^l^	0.866^l^	0.746	0.632	−0.112	0.533	0.220	−0.840^l^	1.000
	*P* value	.042	.036	.036	.026	.088	.178	.833	.036	.675	.036	—

^a^USE: level of usefulness.

^b^SIM: simplicity.

^c^FLE: flexibility.

^d^ACC: acceptability.

^e^SEN: sensitivity.

^f^PVP: predictive value positive.

^g^REP: representativeness.

^h^DQ: data quality.

^i^STB: stability.

^j^TIM: timeliness.

^k^Not applicable.

^l^Correlation is significant at .05 (2-tailed).

^m^Correlation is significant at .01 (2-tailed).

**Figure 2:**
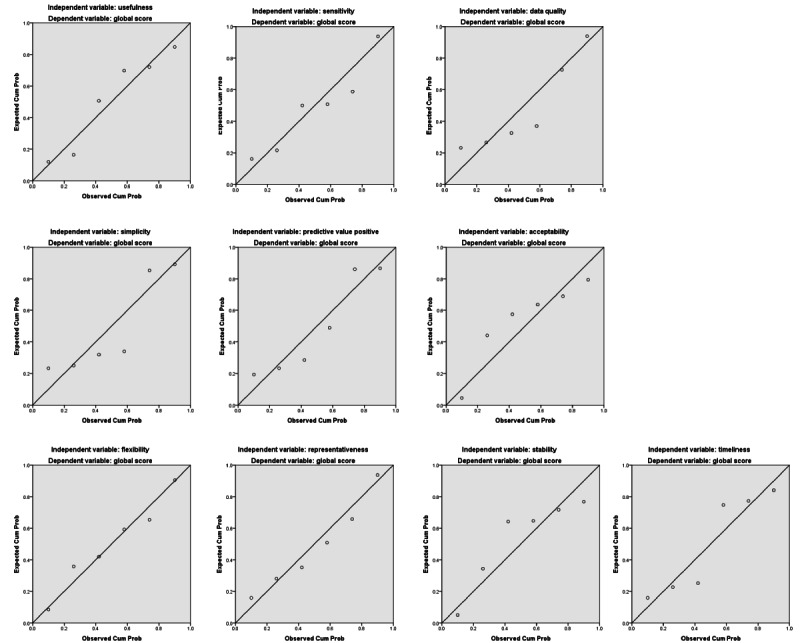
Linear regression between global and each of the attribute scores of Gulf Cooperation Council surveillance systems.

Figure 2 depicts the linear regression analyses between the global score and each of the attribute scores of the GCC surveillance systems. The trends indicate that the global score increases proportionately with the increase in almost all attribute scores. The linearity of the relation is the most apparent with the level of usefulness, flexibility, and acceptability scores.

**Figure 3:**
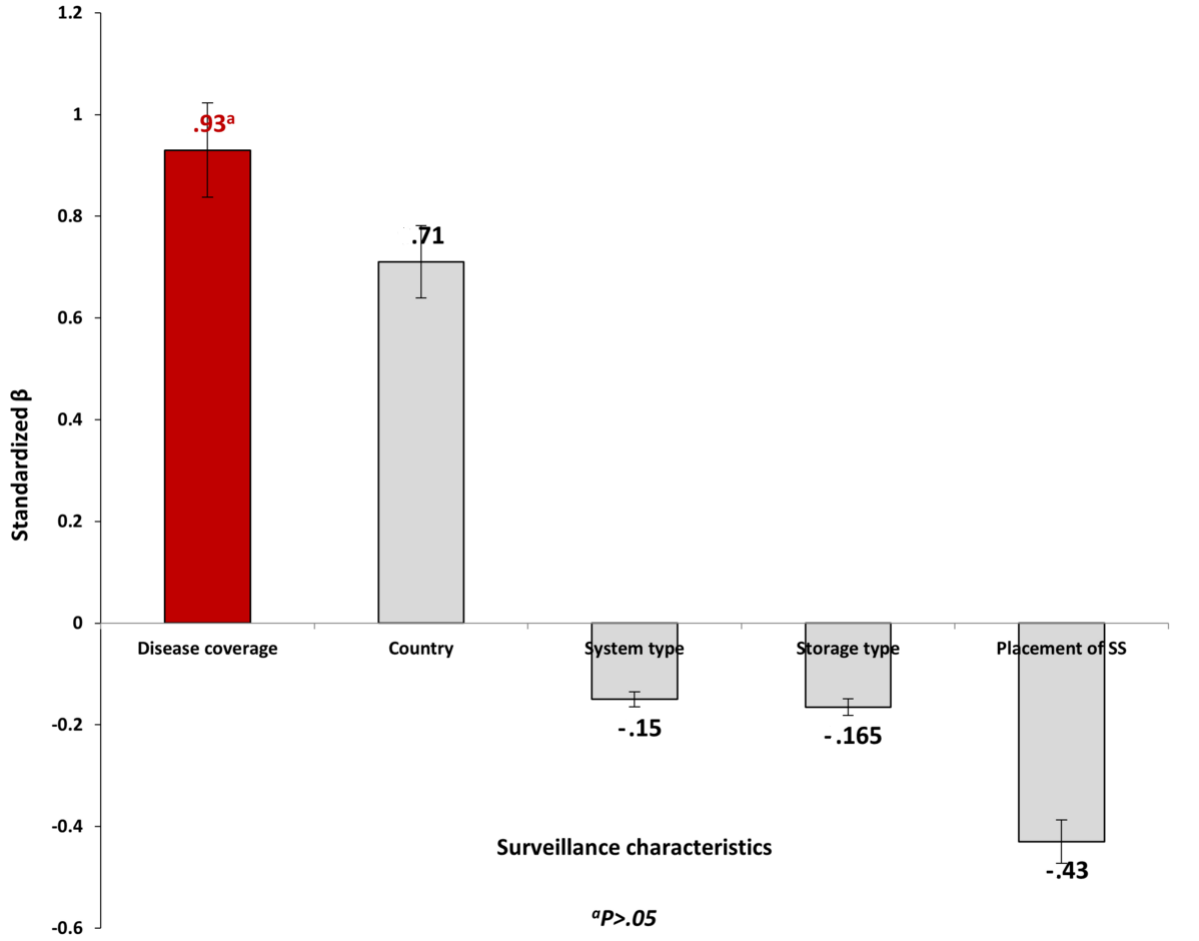
Univariate linear regression modeling of the association between the global score and selected Gulf Cooperation Council public health surveillance characteristics. SS: syndromic surveillance.

Table 7 shows the univariate linear regression model of the association between global scores and the selected characteristics of the GCC public health surveillance system. Surveillance disease coverage was found to be the major significant predictor of GCC surveillance global scores (β=.93; *P*=.006). Country, system type, data storage type, and placement of syndromic surveillance were found to be nonsignificant predictors of the global score (*P*>.05). Figure 3 depicts the pattern of associations between the global scores and the selected characteristics of the GCC public health surveillance system.

**Table 7:** Univariate linear regression modeling of the association between the global score and selected Gulf Cooperation Council public health surveillance characteristics.

Predictor	Regression parameters		
	Unstandardized β (SE)	Standardized β	*P* value
Country	5.06 (2.49)	.71	.11
Disease coverage	9.00 (1.71)	.93	.006^a^
System type	−1.83 (5.98)	−.15	.78
Storage type	−4.25 (12.67)	−.165	.75
Placement of syndromic surveillance	−14.2 (14.6)	−.43	.38

^a^*P*<.05.

## Discussion

### Principal Findings

This study used CDC’s public health surveillance system evaluation guidelines to evaluate the performance of public health surveillance systems in GCC countries regarding their level of usefulness, simplicity, flexibility, acceptability, sensitivity, PVP, representativeness, data quality, stability, and timeliness. It compared the GCC public health surveillance systems’ characteristics with the global score and the 10 system attributes obtained from the CDC evaluation guidelines. In addition, the correlations between the global score and the 10 system attributes were examined. Univariate regression analysis showed that disease coverage is the major predictor of GCC surveillance global scores. This is the first evaluation study with this level of quantitative rigor and geographic magnitude for surveillance systems in the region.

### Comparison With Prior Work

The results of the study demonstrated that the surveillance systems in all GCC countries could detect and monitor CDs and that most of them covered HAIs, especially in Bahrain, the UAE, Saudi Arabia, and Oman. These substantial efforts may be attributed to the adoption of infectious disease surveillance following the World Health Organization’s implementation of the International Health Regulations in 2005. The International Health Regulations mandated that all member states establish the infrastructure needed to detect, monitor, and report CDs to World Health Organization regional offices [12]. Notably, GCC countries do not use surveillance systems to collect administrative data on absenteeism during certain epidemics. This finding contradicts other studies that quantified workplace absenteeism during influenza outbreaks by monitoring sick leave data [13].

However, it must be noted that our data illustrated substantial deficits in the coverage of NCDs across GCC states, with the exception of the UAE and Bahrain. Although NCD monitoring also exists in other GCC countries [14-16], these monitoring mechanisms were siloed and operated independently from the original surveillance systems. As NCDs impose a high economic and social burden on GCC countries [17], incorporating NCDs within the original surveillance systems can potentially consolidate surveillance efforts and aid in prioritizing NCD prevention and control to tackle behavioral and system-level risk factors [18]. Several states in the United States have conducted pilot studies on public health surveillance of chronic diseases and risk factors [19]. Most studies provide a crucial proof of concept; however, obstacles to defining the algorithms and harmonizing them across jurisdictions and countries are still present.

This study demonstrated that the UAE has the highest global score and the highest score in most system attributes, with the widest coverage of diseases among GCC countries. The advancement of the UAE’s health information system is a result of the government’s commitment to deliver the latest digital health infrastructure and solutions to enable proper system monitoring and response [20]. Establishing the region’s first health information exchange within the Emirate of Abu Dhabi is another testament to the country’s commitment [21]. Notably, almost all health systems in the GCC region are undergoing massive system-level reforms, in which overhauls of health information technology, including surveillance systems, constitute a major part [22,23].

Oman’s surveillance system scored the highest in terms of usefulness, simplicity, and flexibility. In 2007, the Ministry of Health in the Sultanate of Oman launched a single, multilayered, and interoperable surveillance platform called the National Electronic Public Health Surveillance System [24]. Repositioning the system to be the single source of reliable data may have contributed toward achieving such high levels of system utility and flexibility. The Saudi Arabian public health surveillance system is undergoing a considerable internal transition, which might account for its suboptimal performance, despite being the largest geographically in the GCC area. This observation is supported by previous studies that extracted data from the upgraded surveillance system in Saudi Arabia [25].

The COVID-19 pandemic, which led to the rapid development of innovative data sets, surveillance technologies, and models, has led to an increased interest in using AI strategies to accomplish public health outcomes [26]. The evidence obtained from this study revealed that only 2 countries were using emerging technologies such as machine learning, AI, and real-time analytics. GCC countries have an immense opportunity to use AI in the rapid identification of populations, interventions, and outcomes of interest for disease surveillance, disease prevention, and health promotion. A Twitter-based influenza detection study, which used a text-mining technique, found that such techniques improved the estimation performance of influenza detection during the peak periods of the disease [27]. Such examples can be leveraged in GCC countries, because we found no explicit evidence of the use of AI approaches in public health surveillance.

In addition to the scientific merit of this study, the findings will aid surveillance administrators and managers in GCC to improve their global score. This is manifested by strong and positive correlations between a country’s global scores and acceptability, flexibility, representativeness, usefulness, and timeliness. Prioritizing these attributes will result in major improvements in global scores and in GCC public health surveillance systems in general. A negative and strong correlation was found between the timeliness and stability of surveillance systems in the GCC, which warrants architects of surveillance systems to design stable and reliable surveillance systems without jeopardizing timeliness and efficiency.

### Limitations

This study was limited by a small sample size, where one expert in the public health surveillance system was purposively assigned to each GCC county to offer the best information regarding the features and functions of the system. However, the use of a relatively adequate sample size and high response rate enabled by the purposive sampling approach strengthened the study’s ability to identify considerable differences. Self-reported questionnaires, however, have the potential to introduce bias, especially with regard to delicate issues such as the usefulness and quality of the system’s data. To better understand the research question and guarantee that the conclusions are grounded in participants’ views and experiences, we suggest conducting a mixed methods evaluation approach, including qualitative research, to evaluate GCC surveillance systems.

### Conclusions

The results of this study demonstrate that GCC surveillance systems appear to be useful and perform sustainably across all countries. These systems have the potential to improve data quality and representativeness. Potential lessons should be leveraged from countries with high scores, especially in using emerging technologies such as machine learning, AI, and real-time analytics. Future research should focus on more comprehensive, mixed methods evaluation approaches for surveillance systems in the GCC. The findings of this study have wider implications for the need for a coordinated GCC surveillance action, which includes capacity building, centralized information exchange, and system architecture redesign to maintain the GCC surveillance systems’ sustainability and responsiveness during upcoming public health challenges.

## Abbreviations

AI: artificial intelligence
CD: communicable disease
CDC: Centers for Disease Control and Prevention
GCC: Gulf Cooperation Council
Gulf CDC: Gulf Center of Disease Prevention and Control
HAI: health care–associated infection
NCD: noncommunicable disease
PVP: predictive value positive
UAE: United Arab Emirates
